# Nursing Process Data for Health Care Cost Prediction Using Machine Learning: Longitudinal Study

**DOI:** 10.2196/93638

**Published:** 2026-09-04

**Authors:** María Consuelo Company-Sancho, Víctor M González-Chordá, María Isabel Orts-Cortés

**Affiliations:** 1 Health Promotion Service, Directorate General for Public Health, Canary Islands Health Service, Las Palmas de Gran Canaria, Canary Islands, Spain; 2 Nursing and Healthcare Research Unit (Investén-isciii), Research Network on Chronicity, Primary Care and Health Promotion (RICAPPS), Carlos III Health Institute, Madrid, Spain; 3 Nursing and Healthcare Research Unit (Investén-isciii), CIBER of Frailty and Healthy Ageing (CIBERFES), Carlos III Health Institute, Madrid, Spain; 4 Nursing Research Group GIENF-241, Nursing Department, Universitat Jaume I, Castellón de la PlanaCastellón, 12071, Spain, 34 964 387744; 5 Department of Nursing (BALMIS), Institute for Health and Biomedical Research (ISABIAL, Group 23), University of Alicante, Alicante, Spain

**Keywords:** standardized nursing terminology, nursing process, nursing diagnosis, machine learning, predictive modeling, health care costs, risk adjustment

## Abstract

**Background:**

Machine learning (ML) has been demonstrated to enhance health care cost prediction by handling high-dimensional data and identifying complex patterns. However, current risk-adjustment models rarely incorporate structured nursing information derived from the nursing process. This information captures care needs and human responses to health problems.

**Objective:**

This study aimed to evaluate the impact of integrating nursing process data into ML-based predictive models of individual health care costs, including cost component analyses, compared with models based solely on sociodemographic, clinical, and morbidity-related variables.

**Methods:**

A retrospective observational study was conducted using a population-based cohort of 1,691,075 individuals aged 15 years or younger who were registered with the Canary Islands Health Service. Predictors were derived from data available up to 2017 and included sociodemographic and clinical variables, Adjusted Morbidity Groups, health care use, and structured nursing records (Functional Health Patterns [FHP], North American Nursing Diagnosis Association [NANDA], Nursing Outcomes Classification [NOC], and Nursing Interventions Classification [NIC]). Predictive models were developed using feedforward neural networks and extreme gradient boosting; predictions were combined using an ensemble approach. An autoencoder was applied as a dimensionality-reduction technique for the nursing variables. Model performance with and without nursing variables was compared on total cost and individual cost components, and the coefficient of determination (*R*²) was used on the test set.

**Results:**

Including the nursing methodology yielded small numerical increases in predictive performance. With respect to total cost, the ensemble model improved the *R*² from 0.5023 to 0.5058 when the nursing variables were added. Although directionally consistent, these gains were limited in magnitude. In the component-level analyses, performance gains were observed in hospital care (*R*²=0.2396) and pharmaceutical costs (*R*²=0.6631). Reducing 789 nursing variables to 16 latent dimensions using an autoencoder substantially simplified the feature space, with predictive performance remaining broadly comparable but without a substantial additional gain.

**Conclusions:**

Integrating structured information from the nursing process is associated with small incremental improvements in ML-based predictive models and complements commonly used sociodemographic, clinical, and morbidity variables. The systematic incorporation of nursing data into predictive tools may contribute to more accurate health care cost prediction and support more holistic, person-centered approaches.

## Introduction

Efficient allocation of resources in health care systems requires accurate and reliable estimates of future expenditure, particularly in a context characterized by budgetary constraints and increasing care complexity. Inaccurate cost estimation may lead to inefficient decision-making and compromise the financial sustainability of health services [1-3]. In this context, machine learning (ML) has gained relevance because it provides tools capable of identifying complex patterns, handling missing values, and improving predictive accuracy compared with traditional statistical methods [4-9].

Within the field of cost prediction, risk-adjustment systems such as Adjusted Clinical Groups (ACGs) [10], Clinical Risk Groups (CRGs) [11], and Adjusted Morbidity Groups (AMGs) [12] play key roles in population risk stratification by anticipating care needs and budgetary requirements. These models have been widely used to estimate costs associated with different patient profiles, and their performance is typically assessed using metrics such as the coefficient of determination (*R*²) [13]. In Spain, the AMG risk-adjustment system explains approximately 31% of the variability in total health care costs, suggesting substantial room for improvement [14]. In response to these limitations, ML techniques have increasingly been applied to risk-adjustment frameworks because of their greater capacity to identify complex patterns in large datasets. The use of neural networks, boosting models, and penalized regression approaches has been shown to improve predictive accuracy [13,15]. Methods such as neural networks and ridge regression have shown good performance in cost estimation using historical data [6].

Moreover, the types of data incorporated into risk-adjustment models have evolved. Electronic health records, which capture sociodemographic data and multiple dimensions of health status, are a rich source of information. Supervised learning models are particularly well suited for analyzing these data, as they can detect latent interactions and manage nonnormally distributed variables, such as health care costs. Leveraging these data allows more accurate estimation of health care resource use and identification of relevant patterns of association [16].

In this context, Bertsimas et al [17] reported that the accuracy of cost prediction models depends largely on the quality, completeness, and standardization of the data used and suggested that incorporating new variables that more faithfully reflect actual care delivery may enhance predictive performance. However, most predictive models, including those based on variables commonly used in ACG, CRG, and AMG risk-adjustment systems, rely primarily on sociodemographic and clinical variables such as gender, age, comorbidities, procedures, and pharmaceutical expenditures [11]. The inclusion of social determinants has been shown to significantly improve the predictive capacity of these models. For example, a recent study reported that incorporating such factors increased the *R*² from 0.327 to 0.388, reducing estimation errors by 3.5 million dollars per 10,000 individuals added [18]. Similarly, factors such as low income, educational level, food insecurity, or housing instability have been significantly associated with high-cost patient profiles [19], although these variables are rarely included in cost prediction models. Harnessing the richness of electronic health record data, together with big data analytics in health care, offers opportunities to improve outcomes and reduce costs [20].

Despite these advances, most predictive models continue to exclude information derived from the nursing process, even though it has the potential to capture essential aspects of health status and service use [2,6]. The incorporation of standardized nursing terminologies such as the North American Nursing Diagnosis Association (NANDA), the Nursing Outcomes Classification (NOC), and the Nursing Interventions Classification (NIC) could substantially enhance predictive models by capturing the impact of nursing interventions. These terminologies, which have been progressively integrated into electronic health records, enable systematic characterization of nursing practice, facilitate assessment of its impact on health outcomes, and support the development of robust predictive models [21]. Understanding and quantifying nursing care in a systematic and effective manner may contribute to optimizing resource allocation and improving health system sustainability [22]. In this context, Functional Health Patterns (FHP) described by Gordon provide a nursing assessment framework that offers a holistic view of patient health, encompassing physical, emotional, and social dimensions. Although this framework may add value in terms of efficiency and resource management, its impact on health care cost prediction has rarely been explored [23]. Nevertheless, a previous study revealed that more than 21% of the variability in total health care costs could be explained by a model including gender, age, and variables derived from the nursing process (NANDA, NOC, NIC, and FHP) [24]. This study adds a comparison between nursing process data and the clinical, sociodemographic, and morbidity-related variables commonly used in cost prediction. It also extends the analysis to partial cost components and explores dimensionality reduction.

Recent literature has highlighted that incorporating additional layers of information, such as social determinants or measures of well-being, significantly improves the performance of health care cost prediction models and helps correct biases affecting vulnerable populations [18,19]. In parallel, the inclusion of structured nursing data has been shown to provide valuable insights into the impact of nursing practice on health outcomes and patient safety [21]. However, to date, no studies have systematically integrated information derived from the nursing process into ML-based models specifically designed for health care cost prediction.

Against this background, leveraging the potential of nursing big data and integrating it with advanced ML techniques represents an opportunity to improve risk-adjustment systems, enhance population stratification, and support more efficient and equitable resource allocation. Accordingly, the aim of this study was to evaluate the impact of integrating nursing process data into ML-based predictive models of individual health care costs, including cost component analyses, compared with models based solely on sociodemographic, clinical, and morbidity-related variables.

## Methods

### Study Design and Setting

The present study forms part of a longitudinal research program based on a single population cohort that aims to understand the determinants of health care costs from a comprehensive perspective. In the first phase, individual health care costs were estimated, and their variability was analyzed according to AMG using multiple linear regression [14]. In the second phase, ML-based predictive models were developed to explain the variability in total health care costs, incorporating information derived from the nursing process [24].

The present (third) phase extends this analytical framework by focusing specifically on the contribution of nursing process variables (NANDA, NOC, NIC, and FHP) to health care cost prediction. To this end, a retrospective, observational, and analytical study was conducted using data from multiple health care information systems. The study was carried out in the Autonomous Community of the Canary Islands (Spain) using data from 2017 and 2018. The predictor variables were derived from data available up to 2017, whereas health care costs were measured for 2018, thus ensuring appropriate temporal ordering for predictive modeling.

### Ethical Considerations

In accordance with the Declaration of Helsinki and Spanish legislation, this study was approved by the Clinical Research Ethics Committee of the Dr. Negrín University Hospital of Gran Canaria (date: January 29, 2021; code: 2021-037-1). All applicable ethical and regulatory principles for studies using secondary health data were observed.

### Study Population

The study population included all individuals aged 15 years or older who were registered in the Canary Islands Health Service health care card database as of December 31, 2017 (N=1,691,075). The entire target population was included. Individuals covered by mutual insurance schemes were excluded; these correspond to public-sector employees whose health care and social care are managed outside the Canary Islands Health Service.

### Variables

Predictor variables were derived from data available up to 2017 and included sociodemographic, clinical, morbidity-related, and nursing process information. The sociodemographic variables comprised age, gender, type of affiliation with the social security system, and the percentage of pharmaceutical copayment. Clinical variables included a Barthel Index score greater than 60, a Pfeiffer test score of 5 or lower, 2 or more hospital admissions in the previous 12 months, and the number of prescribed active pharmaceutical ingredients. Morbidity-related variables were obtained from the AMG system and included the complexity weight and morbidity group.

Nursing process variables were also incorporated and consisted of 44 variables derived from the assessment of the 11 FHP (with 4 possible outcomes per pattern), as well as 211 NANDA nursing diagnoses, 388 NOC outcomes, and 558 NIC interventions. The large number of variables reflects the conceptual structure of nursing data, which captures complementary dimensions of human responses, outcomes, and interventions; therefore, regularization and hyperparameter tuning were applied to mitigate the dimensional burden.

The primary outcome was total health care cost per individual in 2018. This was calculated as the sum of the costs associated with primary care and specialist consultations, emergency department visits, hospital admissions, major ambulatory surgery in public and contracted centers, and pharmaceutical dispensing (Multimedia Appendix 1). In addition, a cost component analysis was conducted for different care settings (primary care, hospital care, and contracted care) and service types (hospital admissions, outpatient consultations, and emergency department visits).

### Data Sources

Data were obtained from the following information systems: the Canary Islands Health Service health care card database (sociodemographic variables), primary care electronic health records (medical and nursing consultations and nursing process variables), hospital electronic health records (hospital consultations and emergency department visits), the Minimum Basic Dataset of the Hospital Activity Registry (hospital admissions and surgical procedures), the Hospital Contracted Care Information System (activity in contracted centers), and the electronic prescribing system of the Canary Islands Health Service (pharmaceutical dispensing). All databases were linked using a unique, encrypted clinical identifier for each individual.

### Data Preprocessing and Analytical Strategy

Prior to analysis, numerical variables (age and AMG complexity weight) were standardized. Cost variables were transformed using natural logarithms to approximate normal distributions. Nursing diagnoses (NANDA), outcomes (NOC), and interventions (NIC) with a prevalence lower than 1 per 10,000 individuals were excluded, as they provided limited information, had a high proportion of zero or missing values, and unnecessarily increased model dimensionality. This threshold was applied pragmatically, as no comparable reference studies were identified, with the aim of filtering infrequent variables and thereby reducing sparsity and computational burden.

After the data were cleaned, a descriptive analysis of the study population was performed. As the entire population was included, no statistical inference techniques were applied. The analytical focus was exclusively on the development and evaluation of predictive models.

Two ML algorithms capable of handling large-scale, high-dimensional health care data were evaluated using complementary modeling approaches. First, a feedforward neural network was implemented [25], which consisted of interconnected layers designed to capture nonlinear relationships and latent patterns. Second, extreme gradient boosting (XGBoost) [26], a gradient-boosted decision tree algorithm recognized for its efficiency, accuracy, and ability to handle missing values and skewed distributions, was applied. Predictions from both models were combined using an ensemble approach based on their averaged outputs to improve robustness [27].

The dataset was randomly split into training (80%), validation (10%), and test (10%) subsets. During model training, the mean squared error (MSE) was used as the optimization metric, while overall performance was assessed using the coefficient of determination (*R*²). Because health care cost data were highly skewed, the outcome was log-transformed before modeling, and both model fitting and performance metrics, including *R*², were calculated on that log-transformed scale. *R*² was used as the primary metric to compare relative model performance across the different predictor sets. Given the very large cohort size, a single training, validation, or test split was considered sufficient for initial model development and evaluation, as both the validation and test subsets each included more than 160,000 individuals. For the feedforward neural network, the final architecture consisted of 4 hidden dense layers (128, 64, 32, and 16 units) with rectified linear unit activation and L1 regularization (l1=0.0000625; l2=0). The model was trained using the root mean square propagation optimizer with a learning rate of 0.0005, a batch size of 64, and a maximum of 60 epochs. Early stopping was applied based on the validation *R*² (monitor=val_r_square, mode=max, and patience=5), and the best model weights were restored. For XGBoost, the final model was trained with a learning rate of 0.0960, a maximum tree depth of 10,500 estimators, a subsample of 0.9413, full column sampling per tree (colsample_bytree=1.0), L1 regularization (reg_alpha=10.0), and L2 regularization (reg_lambda=0.001). Hyperparameter optimization was conducted on the validation set, and the final model performance was evaluated exclusively on the test set. A fixed random seed of 42 was used to ensure reproducibility.

The analytical strategy involved 3 stages. First, baseline models excluding the nursing process variables and extended models including these variables were developed and compared for total health care cost prediction. Second, cost component analyses were conducted at both the care setting level (primary care, hospital care, and contracted care) and the service level (hospital admissions, outpatient consultations, and emergency department visits), and model performance was compared with and without the nursing process variables. Third, an autoencoder was applied to reduce the dimensionality of the nursing process variables, as the feature block was high-dimensional and predominantly binary, making a more flexible nonlinear approach preferable to the conventional principal component analysis. This neural network architecture compresses high-dimensional input data into a lower-dimensional latent representation and reconstructs it, thereby enabling the identification of unsupervised latent patterns [28]. Autoencoders have previously been applied in hospital cost prediction studies [29]. All analyses were performed using Python version 3.10.8, with the *pandas*, *NumPy*, *scikit-learn*, *TensorFlow*, and *XGBoost* libraries.

## Results

### Characteristics of the Study Population

The study included 1,691,075 individuals aged over 15 years, including 863,071 (51.0%) women; the mean age was 46.6 (SD 17.9) years, and 17.4% (n=294,077) were older than 65 years. The mean AMG index weight was 5.82 (SD 5.89) points, which was higher in women (6.25, SD 6.06) than in men (5.10, SD 5.62) and increased with age (Spearman ρ=0.47). A total of 216,630 (12.8) women and 175,536 (10.4) men were pensioners. The mean number of prescribed medications was 4.35 (SD 5.10), with higher values observed in women.

Overall, 22,800 (1.3%) individuals had 2 or more hospital admissions in the previous year, 6308 (0.4%) had a Barthel Index score greater than 60, and 7271 (0.4%) demonstrated cognitive impairment (Pfeiffer score≥5). Nursing records (FHP, NANDA, NOC, and NIC) were present for 57.9% (980,437/1,691,075) of the population, although only 2.7% (46,380/1,691,075) had completed the full nursing process. On average, 3.84 (SD 3.58) FHP were assessed per person, together with 3.07 (SD 2.9) NANDA nursing diagnoses, 2.77 (SD 2.96) NOC outcomes, and 4.12 (7.04) NIC interventions.

The mean cost per patient was €1283.08 (SD €3007; €1=US $1.19 as of December 31, 2017), with higher values observed among women. Hospital admissions and consultations accounted for the highest cost components. Costs were heterogeneously distributed across care settings, with those for consultations (mean €355.07, SD €586.87) and pharmaceutical dispensing (mean €345.53, SD €946.09) being especially notable. Table 1 presents details of the variables included in the models; the complete cohort profile can be found in previous publications [14,24].

**Table 1.** Characteristics of the study population and variables included in the predictive models.

Variables	Total^a^	Women	Men
Sociodemographic and clinical, n (%)			
Pensioners	392,166 (23.2)	216,630 (12.8)	175,536 (10.4)
≥2 hospital admissions	22,846 (1.4)	12,082 (0.7)	10,764 (0.6)
Barthel Index <60	5971 (0.4)	4315 (0.3)	1656 (0.1)
Pfeiffer score ≥5	6834 (0.4)	5178 (0.3)	1656 (0.1)
AMG^b^ system, mean (SD)			
Complexity weight	5.82 (5.89)	6.25 (6.06)	5.10 (5.62)
Mean number medications	4.35 (5.10)	5.19 (5.47)	3.48 (4.55)
Nursing process, n (%)			
Nursing process records	980,437 (57.9)	533,231 (61.8)	447,206 (54.0)
Completed nursing process	46,380 (2.7)	29,405 (3.4)	16,975 (2.1)
At least 1 FHP^c^ assessed	415,068 (24.5)	247,162 (28.6)	167,906 (20.3)
At least 1 active NANDA^d^	916,024 (54.2)	489,738 (56.7)	426,286 (51.5)
At least 1 active NOC^e^	772,730 (45.7)	419,496 (48.6)	353,234 (42.7)
At least 1 active NIC^f^	787,510 (46.6)	427,370 (49.5)	360,140 (43.5)
Cost (€; €1=US $1.19)^g^, mean (SD)			
Mean total health care cost	1283.08 (3007)	1377.04 (2868.27)	1185.14 (3142.05)
Hospital admissions	463.11 (2207.39)	461.55 (2060.64)	464.72 (2350.62)
Outpatient consultations	355.07 (586.87)	418.80 (623.38)	288.64 (538.24)
Emergency department visits	119.35 (324.68)	131.10 (332.68)	107.09 (315.67)
Pharmaceutical dispensing	345.53 (946.09)	365.55 (914.18)	324.67 (977.81)
Hospital care	571.90 (2274)	597.73 (2118.51)	544.99 (2425.37)
Primary care	246.85 (421)	290.96 (454.43)	200.87 (378.69)
Contracted care	118.8 (808)	122.76 (827.41)	114.59 (786.31)

a The cohort included 1,691,075 individuals (863,071 women and 828,004 men).

b AMG: Adjusted Morbidity Groups.

c FHP: Functional Health Pattern.

d NANDA: North American Nursing Diagnosis Association.

e NOC: Nursing Outcomes Classification.

f NIC: Nursing Interventions Classification.

g Health care cost components are presented by care setting (primary care, hospital care, and contracted care) and by service type (hospital admissions, outpatient consultations, and emergency department visits), as well as pharmaceutical dispensing.

### Predictive Performance of the Baseline and Extended Models for Health Care Cost Prediction

The neural network architecture with the best performance was a feedforward network with 4 hidden layers (128, 64, 32, and 16 nodes) and an output corresponding to the logarithm of the total cost. For the XGBoost model, Bayesian hyperparameter optimization was applied to identify the combinations with the best performance, and an ensemble model was subsequently generated by averaging the predictions.

**Figure 1.**
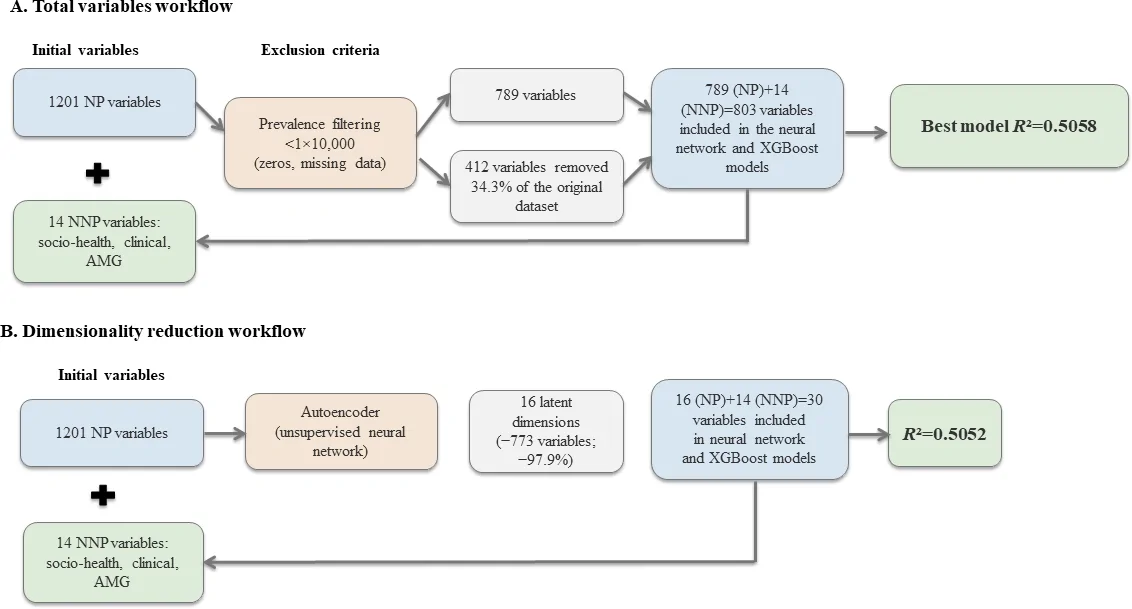
Workflow of nursing-variable selection and dimensionality reduction for the neural network and XGBoost models. AMG: Adjusted Morbidity Group; NNP: nonnursing process; NP: nursing process; XGBoost: extreme gradient boosting.

The initial feature set included sociodemographic and clinical variables, together with 1201 nursing process variables. After those with a positive prevalence of fewer than one per 10,000 patients were excluded, 789 nursing variables and 14 sociodemographic, clinical, and AMG system variables were retained (Figure 1).

In the baseline models, excluding the nursing variables, the *R*² was 0.4996 for the neural network, 0.5030 for XGBoost, and 0.5023 for the ensemble model. After the nursing process variables were incorporated, the predictive performance increased to 0.5026 (MSE 3.8561), 0.5065 (MSE 3.7936), and 0.5058 (MSE 3.7981), respectively, indicating improvements across all 3 algorithms.

### Predictive Performance by Cost Component According to Care Setting and Service Type

Across most cost components, the inclusion of the nursing process variables improved predictive performance, with variations depending on the care setting and model type (Table 2). For hospital care, the greatest increase was observed in the ensemble model, with *R*² increasing from 0.2161 to 0.2396 (+0.0235), while the improvements in the neural network and XGBoost models were similar (+0.0230 and +0.0223, respectively). For primary care, improvements were more moderate but consistent; the ensemble model increased from 0.4388 to 0.4442 (+0.0054), and comparable gains were observed for the neural network (+0.0050) and XGBoost (+0.0054). For emergency department visits, the predictive performance also increased, with that of the ensemble model increasing from 0.1431 to 0.1506 (+0.0075) and similar gains observed for XGBoost (+0.0074). For contracted care, improvements were more limited in magnitude but were observed across all models, with the ensemble model’s performance increasing from 0.0389 to 0.0443 (+0.0054).

**Table 2.** Predictive performance (*R*²) of machine learning–models with and without nursing process variables across health care cost components.

Health care cost components and models	No (*R*²)	Yes (*R*²)	Differences
Care settings			
Primary care			
Neural network *R*²	0.4359	0.4409	+0.0050
XGB^a^ *R*²	0.4394	0.4448	+0.0054
Ensemble *R*²	0.4388	0.4442	+0.0054
Hospital care			
Neural network *R*²	0.2133	0.2363	+0.0230
XGB *R*²	0.2163	0.2386	+0.0223
Ensemble *R*²	0.2161	0.2396	+0.0235
Contracted care			
Neural network *R*²	0.0382	0.0402	+0.0020
XGB *R*²	0.0380	0.0452	+0.0072
Ensemble *R*²	0.0389	0.0443	+0.0054
Service types			
Hospital admissions			
Neural network *R*²	0.0624	0.0618	−0.0006
XGB *R*²	0.0636	0.0652	+0.0016
Ensemble *R*²	0.0641	0.0650	+0.0009
Outpatient consultations			
Neural network *R*²	0.4568	0.4608	+0.0040
XGB *R*²	0.4606	0.4652	+0.0046
Ensemble *R*²	0.4598	0.4642	+0.0044
Emergency department visits			
Neural network *R*²	0.1407	0.1473	+0.0066
XGB *R*²	0.1434	0.1508	+0.0074
Ensemble *R*²	0.1431	0.1506	+0.0075
Other cost components			
Pharmaceutical costs			
Neural network *R*²	0.6565	0.6612	+0.0047
XGB *R*²	0.6588	0.6631	+0.0043
Ensemble *R*²	0.6584	0.6631	+0.0047

a XGB: extreme gradient boosting.

When analyzed by service type, outpatient consultations and emergency department visits showed the most consistent improvements. For outpatient consultations, the *R*² of the ensemble model increased from 0.4598 to 0.4642 (+0.0044), whereas for emergency department visits, it increased from 0.1431 to 0.1506 (+0.0075). For hospital admissions, the changes were minimal; the ensemble model *R*² increased slightly from 0.0641 to 0.0650 (+0.0009), whereas that of the neural network marginally decreased (−0.0006).

### Predictive Models Using Latent Variables Derived From the Autoencoder

Finally, an autoencoder was applied to reduce the 789 nursing variables to 16 latent dimensions while preserving relevant data patterns. These compressed variables were incorporated with the sociodemographic, clinical, and AMG-related variables. With this combination, the models achieved *R*² values of 0.5013 for the neural network, 0.5062 for XGBoost, and 0.5052 for the ensemble model. Thus, dimensionality reduction markedly simplified the nursing feature space while yielding predictive performance that was broadly comparable to that of the models using the larger nursing-variable set, but without a substantial performance improvement.

## Discussion

### Principal Findings

The integration of variables derived from nursing methodology into ML-based prediction of total and component-level health care costs resulted in small numerical improvements in total cost prediction, whereas changes observed across cost components were heterogeneous and, in many cases, of small magnitude. These results suggest that nursing information may contain an additional predictive signal, although the magnitude of this contribution was limited, thereby reflecting differences in care delivery structures and in the relationship between care processes and resource use. Accordingly, the balance between added model complexity and predictive benefit should be interpreted with caution, as the contribution of nursing information varies according to the care setting and service type considered.

To date, the predominant methodologies used in health care cost analysis have systematically excluded data related to nursing interventions and outcomes, despite evidence that nursing terminologies can improve both the quality and the efficiency of resource management [30]. Their standardization enables the documentation and quantification of nursing work and provides a holistic view of the patient that is not usually captured by traditional biomedical data. Recent studies have shown that incorporating nursing information into clinical databases facilitates a better understanding of the impact of nursing practice on patient outcomes [21] and contributes to improving patient safety [31], with a potential indirect effect on cost reduction.

However, the literature on the application of ML to nursing data has focused primarily on the development of intelligent clinical tools, such as virtual assistants, fall prediction models, and patient monitoring systems, rather than on economic outcomes [32]. In this context, the present study represents, to our knowledge, a novel approach by systematically integrating nursing process information into cost prediction models using ML techniques, thereby extending previous research in which the added economic value of nursing data has rarely been evaluated.

The use of ML algorithms has demonstrated usefulness in health care cost prediction by overcoming limitations inherent in traditional statistical models, such as data skewness and distributional bias [6,33]. In general, the variables most commonly used to predict health care expenditure are medical diagnoses and prescribed medications [2,34]. For example, in the study by Drewe-Boss et al [2], neural network–based models relied primarily on medical diagnoses coded according to the *International Classification of Diseases*, 10th Revision (ICD-10) and the Anatomical Therapeutic Chemical (ATC) classification system. In contrast, models built using ridge regression placed greater weight on Gebührenordnungspositionen codes, which are broadly comparable to the diagnosis-related groups used in Spain and other European countries.

Similarly, comorbidity may be a better predictor of pharmaceutical consumption and other health care products than clinical complexity, which encompasses psychological, social, and functional dimensions [35]. The findings of the present study are partially consistent with this pattern because the inclusion of nursing variables increased the predictive capacity for total health care costs, although the magnitude of this increase was modest. Nevertheless, given the limited magnitude of the observed improvement, its operational relevance for population risk adjustment or resource planning should be interpreted with caution.

Health care costs are complex and influenced by multiple factors. The incorporation of measures of well-being into prediction models has been shown to improve the estimation of future health care costs by 5.7% to 13% [36]. Another study revealed that each one-point increase in well-being indices was associated with a 1% reduction in health care expenditure [37]. Similarly, the inclusion of social determinants in predictive models has demonstrated additional benefits: in populations characterized by high levels of poverty, inequality, or lack of coverage, social determinants reduced cost underestimation and improved predictive performance by 3%, corresponding to approximately US $200 per person per year [18].

The integration of this type of data into ML models represents a promising avenue for improving the efficiency and sustainability of health care systems [38]. Along these lines, nursing data capture dimensions related to functional status, care needs, and human responses to health problems that are traditionally absent from cost models and could complement the social and clinical determinants commonly used.

In the analyses performed, pharmaceutical costs showed the highest predictive accuracy, with *R*² values increasing from 0.6584 to 0.6631 following the inclusion of nursing variables. This finding suggests that these variables may capture information related to therapeutic adherence, medication management, and educational processes, which are consistent with the role of nursing practice. Although the observed improvement was modest, its limited magnitude means that any interpretation regarding its practical usefulness should remain cautious. In contrast, in other areas, such as outpatient consultations or primary care, changes were small and, in some cases, showed a downward trend, indicating a limited effect of nursing variables in these specific settings. Overall, these patterns indicate that the contribution of nursing methodology is not uniform and may be more relevant in functions closely linked to clinical follow-up and pharmacological management.

In primary care, a setting in which nursing plays a central role, particularly in the management of chronic conditions, an improvement in predictive performance was also observed (*R*² increasing from 0.4388 to 0.4442). This result suggests that long-term care and continuous follow-up, areas with greater nursing involvement, may benefit more from the systematic incorporation of the nursing process into predictive models.

In contrast, the XGBoost models consistently showed better predictive performance than the neural networks in the present analysis, although the literature has reported mixed findings. For example, in a study on pharmaceutical cost prediction, boosted tree–based models outperformed neural networks (area under the curve [AUC]=0.74) [39], whereas another study on hospital cost prediction reported better performance for neural networks (*R*²=0.813) than for decision trees (*R*²=0.713) [40]. Nevertheless, XGBoost is widely regarded as one of the most robust algorithms for tabular data and for scenarios with missing values [41]. The consistency of its performance in the present study further supports its suitability for complex health care data with multiple categorical and sparse variables, such as those derived from the nursing process.

One of the main uses of predictive models lies in identifying relevant features within large datasets and in understanding their contribution to outcome prediction. However, when the number of variables is high, the presence of irrelevant or redundant dimensions may adversely affect model performance by introducing noise and unnecessarily increasing complexity. In the present study, the modeling strategy was designed to assess the incremental predictive value of the nursing data block as a whole, rather than to determine the individual relevance of specific nursing diagnoses, outcomes, interventions, or functional patterns.

In this context, autoencoders have become established as effective tools for dimensionality reduction, redundancy elimination, and the generation of latent representations that preserve essential information [42]. In the present study, autoencoder implementation enabled the condensing of 789 nursing methodology variables into 16 latent variables. Although this reduction preserved relevant patterns and substantially simplified model complexity, it did not translate into a substantial improvement in predictive performance, indicating that dimensionality reduction alone was not sufficient to strengthen predictive performance in a meaningful way. Therefore, the autoencoder should be interpreted here as a strategy for representation simplification rather than as a predictive advantage. Future studies could explore which of these latent dimensions are most informative and whether they capture distinct patient profiles.

Among the most widely used health care management tools are risk-adjustment models such as ACGs, Diagnosis-Related Groups, and AMGs, which are extensively applied in Spain and other countries. These models are integrated into electronic health records with the aim of classifying morbidity, predicting hospital admissions or mortality, and estimating health care costs, thereby supporting efficient resource allocation and informing managerial decision-making. However, because they are largely based on linear statistical approaches, they present important limitations when applied in settings characterized by complex, nonlinear, and high-dimensional data, as is common in clinical information systems [15]. These limitations may result in the underestimation of actual health care costs and reduced predictive accuracy in scenarios of high care complexity.

In contrast, ML algorithms have been shown to outperform traditional models across several key metrics. For example, in mortality prediction, they achieve AUC values ranging from 0.92 to 0.94, compared with 0.91 for classical models, and in the prediction of hospital readmissions, performance improves from 0.69 to 0.75-0.76 [43]. In light of these findings, the present results suggest that combining ML algorithms with variables derived from the nursing process could increase the precision and practical use of current risk adjustment models, particularly in settings characterized by a high nursing workload.

### Implications for Practice

The findings of the present study indicate that the systematic incorporation of variables derived from the nursing process into clinical information systems may contribute to improving the accuracy of health care cost prediction models and could enhance population stratification tools. Integrating these data may enable more accurate identification of patient groups with greater resource needs and facilitate the planning of more efficient, evidence-based interventions.

In addition, recognizing the predictive value of nursing documentation highlights its contribution to health care system management and underscores the importance of promoting structured, interoperable, and high-quality nursing records in clinical practice.

### Strengths and Limitations

The present study has several limitations that should be considered when interpreting the findings. First, the observation period was limited to a single year, which restricts the ability to analyze long-term cost trajectories. Although previous studies have indicated that predictive performance improves with longer follow-up periods [2,44], the use of a large population-based cohort (N=1,691,075) provides a robust foundation and reduces the risk of overfitting, thereby strengthening the external validity of the analysis.

Second, comparability with other studies is limited because of the heterogeneity in the algorithms, validation strategies, and performance metrics reported in the literature. In addition, no studies have been identified that have systematically integrated nursing process variables into health care cost prediction models, which hinders direct comparison but also highlights the innovative nature of this work.

Another limitation relates to dimensionality reduction using autoencoders. Although this approach enabled the synthesis of 789 nursing variables into 16 latent components, the clinical interpretability of these components was lower, and no substantial improvement in predictive performance was observed. Nevertheless, these latent representations offer opportunities for future research aimed at identifying patient profiles and patterns associated with resource use.

Finally, the rapid evolution of AI techniques means that some approaches may become outdated over time. However, the methods applied represent well-established standards in ML and are appropriate for the stated objectives. Replicating the present study in other health care systems would help strengthen the generalizability of the findings and broaden their applicability.

### Conclusions

The incorporation of variables derived from the nursing process into ML-based health care cost prediction models was associated with small incremental improvements in the explanatory capacity for total health care costs, whereas effects observed across cost components were heterogeneous and of smaller magnitude. These findings suggest that structured nursing information may complement the sociodemographic, clinical, and morbidity-related variables commonly used in health care expenditure estimation.

The results highlight the relevance of moving toward the integration of nursing process information into population-stratification systems and risk-adjustment models, which are key tools for health care planning and efficient resource allocation. The inclusion of dimensions related to functional status, care needs, and human responses broadens the traditional perspective based on clinical and administrative data, thereby supporting more comprehensive approaches to cost prediction.

The potential of ML to improve the estimation of health care resource use should not be limited exclusively to conventional biomedical predictors. The incorporation of structured information derived from nursing practice may enhance the understanding of the determinants of health care costs and support the development of more holistic, person-centered predictive models aligned with contemporary approaches to integrated care.

## Supplementary material

10.2196/93638 Multimedia Appendix 1 Cost variables used to calculate total costs.

## Abbreviations

ACG: Adjusted Clinical Group
AMG: Adjusted Morbidity Group
ATC: Anatomical Therapeutic Chemical
AUC: area under the curve
CRG: Clinical Risk Group
FHP: Functional Health Patterns
ICD-10: *International Classification of Diseases, 10th Revision*
ML: machine learning
MSE: mean squared error
NANDA: North American Nursing Diagnosis Association
NIC: Nursing Interventions Classification
NOC: Nursing Outcomes Classification
XGBoost: extreme gradient boosting
