# Supplementary material for: Nursing Process Data for Health Care Cost Prediction Using Machine Learning: Longitudinal Study
Source: JMIR Nurs. 2026 Sep 4;9:e93638. doi: 10.2196/93638 (PMC13544411; doi:10.2196/93638)
Supplement: Supplementary file 1 [file nursing-v9-e93638-s001.docx]

**Appendix 1**

**Cost variables used to calculate total costs:**

Medical consultations in the primary care centre (€28.78/consultation) [COSTE_AP_MED_CEN_18]

Home primary care medical consultations (€43.66/consultation) [COSTE_AP_MED_DOM_18]

Nursing consultations in the Primary Care centre (€26.62/consultation) [COSTE_AP_ENF_CEN_18]

Home primary care nursing consultations (€106.47/consultation) [COSTE_AP_ENF_DOM_18]

Primary care midwifery consultations (€59.32/consultation) [COSTE_AP_MAT_18]

Social worker consultations in primary care (€176.24/consultation) [COSTE_AP_TS_18]

Outpatient medical emergencies (PC) in public centres (€28.78/consultation) [COSTE_AP_URG_MED_18]

Outpatient nursing emergencies (PC) in public centres (€26.62/consultation) [COSTE_AP_URG_ENF_18]

Hospital emergencies in public centres (€227.78/episode) [COSTE_AE_URG_18]

Specialised care medical consultations as a first visit in public centres (€137.22/consultation) [COSTE_AE_CONS_PRIMERAS_18]

Successive specialised care medical consultations in public centres (€82.33/consultation) [COSTE_AE_CONS_SUCESIVAS_18]

Emergencies in state-subsidised private centres [COSTE_CC_URG_18]

Specialised care medical consultations in state-subsidised private centres [COSTE_CC_CONS_18]

Physiotherapy treatments in state-subsidised private centres [COSTE_CC_FISIO_18]

Rehabilitation consultations in state-subsidised private centres [COSTE_CC_REHAB_18]

Hospitalisations and outpatient major surgery. In these cases, a standard cost per epi-sode is used, based on the applicable related diagnostic group. (GDR-AP):

Hospitalisations in public centres [COSTE_AE_HOSP_18]

Home hospitalisations in public centres (HADO) [COSTE_AE_HADO_18]

Major outpatient surgery in public centres [COSTE_AE_CMA_18]

Hospitalisations in private centres (including private and state-subsidised private ac-tivity) [COSTE_CC_HOSP_18]

Major outpatient surgery in private centres (including private and state-subsidised private activity) [COSTE_CC_CMA_18]

Prescriptions dispensed in pharmacies of the Canary Islands Health Service, whether electronic or on paper. [COSTE_REC_DISP_18]

Some costs without available economic information could not be included. For instance, patient transportation costs, hemodialysis, orthotic and prosthetic benefits, oxygen therapy, or hospital-dispensed pharmacy services, among others, were not considered.
